# Parental Knowledge, Preference and Needs of Child-Rearing Family Programmes: A Case in Chinese Inner Mongolia Minority Region

**DOI:** 10.3390/ijerph20010434

**Published:** 2022-12-27

**Authors:** Jinjin Lu, Yi Huang, Jian Chen

**Affiliations:** 1 Academy of Future Education, Xi’an Jiaotong-Liverpool University, Suzhou 215000, China; 2 Department of Psychology, Faculty of Social Studies, Masaryk University, 60200 Brno, Czech Republic

**Keywords:** child rearing, parental knowledge, parents’ rearing preferences and needs, child mental and physical development, minority region

## Abstract

Core parenting knowledge is critical for enhancing children’s physical and mental development throughout the early stages of life, and it is essential to understand parents’ preferences and needs in acquiring core parenting knowledge. In particular, with the launch of the Family Education Law in China, parents, community workers, and early childhood (EC) teachers gather together to engage in scientific and evidence-based programmes. However, Chinese historical and cultural factors, such as parents’ authority, family structure, child rearing, and non-scientific programme support, affect the improvement of parents’ knowledge and understanding of child-rearing programmes. This study used a qualitative research method to investigate parents’ knowledge, preferences, and needs regarding the potential implementation of interdisciplinary child-rearing programmes in the Inner Mongolia region of China. In total, 24 participants volunteered to take part in the study. The results reveal that most participants were positive and eager to acquire knowledge using evidence-based information to assist children’s mental and physical development. However, parents often have mixed opinions on obtaining knowledge and skills to enhance children’s academic and soft skills in the context of traditional Chinese cultural norms. Suggestions and implications are also provided for parents, social workers, EC teachers, and policymakers for future research.

## 1. Background

Parents are generally believed to be the most important influences on the early stages of a child’s life. From birth, children mainly rely on their parents’ knowledge and perceived experiencing of raising them until they grow up. In this long process, parents and caregivers protect and care for young children and provide the best opportunities for them in their life trajectory, which promotes their overall well-being [1] though parents may appear to have a knowledge of children’s characteristics, many still lack knowledge or do not have sufficient support from family programmes that address how best to understand young children. Without sufficient support and successful family programmes, parents are confronted with uncertainties and challenges to ensure their child’s physical and mental development in their early years, and particularly for those in vulnerable communities [2].

Parent education family programmes began in the early 1930s [3] and were soon legislated in the USA [4]. Their purpose is to provide a family with child development information and encourage effective parenting skills. Parenting programmes are one of the most important methods for enhancing parental knowledge, providing parents with resources, and supporting parents’ ability to better understand their children [5]. The first five years of a child’s life is crucial for their physical, emotional, social, and intellectual development.

In recent years, parents have obtained knowledge and information for their young children from various family education programmes. For example, it was reported that parent education could be an effective way to strengthen families and prevent child maltreatment [6]. In the learning process, parents could obtain basic skills of intervention and improve their understanding of related research evidence and results.

Effective and supported programmes are responsive to the unique needs of parents and consider the preferences of a variety of social groups to ensure positive outcomes [7,8,9,10]. The unique needs and preferences of parents regarding the content of the programmes depend on their life situations [5]. For example, parents who are from minority regions and are of a low socioeconomic status are more likely to learn information and acquire skills regarding a child’s safety and security than those who have a higher socioeconomic status. However, little has been discussed regarding parental needs and their preferences for using evidence-based information in child rearing, both in the West and in China, particularly from the perspectives of parents. Questions concerning issues such as parents’ preferences for obtaining the resources and required information in a family supported program, and parents’ understanding of the adoption of interdisciplinary teamwork in child-rearing programmes has not previously been explored by researchers. All of these questions should be analysed and discussed for a modern family structure in a Chinese context.

### 1.1. Theoretical Framework

This study is underpinned by Bronfenbrenner’s [11] ecological theory, which provides a framework for examining parents’ perceptions of their needs. Based on ecological theory, individuals need to interact with others in the human development process, and are situated within a changing and dynamic living environment, rather than in a stable status quo [11]. Parenting experience might be a result of interactions with other family members, community officers, and other social organisations. The family environment “consists of the totality of the physical, biological, social, economic, political, aesthetic, and structural surroundings for human beings and the context for behaviour and development” [12]. From an ecological perspective, interactions with others and the interdependence of humans (as individuals, groups, and societies) within the environment are vital. In the dynamics of the ecological system, family income, relationships, parents’ workplaces, and neighbourhood all have the potential to change, with humans needing to adapt to their new environment. In this process, parents’ needs and preferences may also differ when the system has been developed. Chung [13] found that positive experiences in communication and interactions among parents could increase their likelihood of involvement in family education and vice versa. Chung [13] argued that there is a significant gap between the family education programmes designed for EC teachers/community workers and parents’ needs in their daily lives. Similar research results were found in [14], which claimed that immigrants and disadvantaged parents need further understanding by stakeholders before designing family programmes in the local social context.

Parents’ needs and preferences are not only obtained from one professional team but also advocated by interdisciplinary professionals [15]. Bronfenbrenner’s model is not a model of a family process or family development per se but provides a framework for examining the ways that interfamilial processes are influenced by both extra-familial conditions and the environment. It is undoubtedly true, therefore, that the family education programme is a systematic and complex project that requires different professionals to contribute efforts and work in an interdisciplinary manner.

### 1.2. Previous Studies on Parental Knowledge, Preferences and Needs

For several decades, Western studies have shed light on parental knowledge [16]. Breiner et al. [16] clarified that the term ‘knowledge’ refers to facts, information, and skills gained through experience or education and the understanding of an issue or phenomenon. Benasich and Brooks-Gunn [17] defined it as the understanding of “developmental norms and milestones, processes of child development, and familiarity with caregiving skills” (p. 1187). Parental knowledge is thought to encourage successful cognitive development in children and attend to their needs at every stage of growth [18]. A large number of quantitative studies have either focused on investigating a range of key factors or parents’ beliefs and attitudes that would influence parental knowledge and their understanding of child rearing. These include aspects of, for example, maternal health, age, parents’ educational background, and family socioeconomic status [19,20,21,22]. Other researchers have used longitudinal studies to examine the associations between parental knowledge and monitoring to reduce children’s risks [23,24]. Although the core aspects described are needed to consider the range of parenting knowledge, we believe that the manner in which parents identify the parenting training and family programmes needed to obtain these skills is far more significant. In this paper, we intend to explore parental knowledge from the standpoint of parents’ current knowledge and what they are able to gain from potential parental family education programmes.

Researchers generally believe that parental knowledge is a decisive factor that leads to different parental beliefs, practices and child development. However, parents’ needs and preferences in obtaining information and knowledge might differ for historical and geographical reasons, particularly in marginalized and vulnerable groups. For example, Safadi et al. [22] examined Jordanian mothers’ knowledge of infants’ child-rearing practices to better provide sources of information. The research results show that maternal knowledge is limited to children’s security and safety, with little knowledge pertaining to cognitive and interactive skills with children. They proposed that nurses need to systematically assess parents’ needs for health education, including physical as well as cognitive, emotional, and parent–infant interaction materials. In this view, it is essential for us to know parents’ needs and to address them at the outset. Without knowing parents’ needs and preferences, it is difficult to design relevant educational materials to improve their knowledge and skills across these domains.

Most studies examine parents’ preferences and needs via a quantitative research paradigm [5,25,26,27]. For example, Kim [26] adopted cluster analysis to examine parenting education interests, family characteristics, and preferred delivery methods of parenting education among parents or caregivers of 0–5-year-old children in Southern Nevada. The survey analysis shows that some topics were strongly favoured by parents, such as “[learning about] fun ways to share books with my children to help them succeed when they get to school,” “[learning] tips to keep my child safe and healthy,” and “[discovering] inexpensive activities that will help my child learn and develop” [26]. Similarly, Nathans et al. [27] made use of surveys to investigate how pre-service teachers identified and understood parents’ knowledge and involvement in education programmes. They found that parents’ involvement in family education programmes is essential, but that they should be varied and tailored to ethnic groups and geographical sites. Scholars have proposed that case studies could be adopted to promote a pre-service of appropriately representing teachers’ thoughts on practices and partnerships across varied geographic and cultural settings [27].

To the best of our knowledge, very few scholars have used a qualitative research method to study parents’ needs and preferences to obtain skills through supported family programmes. Ansari’s study [14], which is an exception, used four focus groups to explore the characteristics of 30 Latino immigrant parents whose children were enrolled at a state-funded preschool in the United States. The results show that parents’ and families’ needs and concerns are far more significant than other factors that could influence parents’ decision-making processes in receiving resources for pre-school education. Similarly, Aloola et al. [28] conducted semi-structured interviews to investigate parents’ needs and preferences regarding support for students with asthma in Saudi Arabian primary schools. The results show that most participants did not believe that teachers could deal with asthma if a student were to experience an asthma attack. They proposed that staff should be provided training and that more school community engagement is urgently needed to improve the management of asthma in Saudi Arabian schools.

In summary, within the small number of studies focusing on examining parents’ needs and preferences, quantitative research methods are the most popular for analysing parents’ needs and comparing parents’ choices with existing inferential measurements. In this way, researchers can understand the necessities of parents’ needs in a particular social context. However, these inferential measurements were mainly developed in the West, and thus are tailored for the population in a Western social welfare system and may not be suitable for the Chinese cultural welfare system. Moreover, a large amount of numerical data from surveys could not be representative of vulnerable groups and people in a minority group, which is needed for further exploration.

### 1.3. Parent Education Family Programmes in a Chinese Cultural Context

Parent education family programmes (called family education programmes in mainland China) have varied in form and are presented differently across geographical areas due to unbalanced historical, social, economic, and political development [29]. In China, the family education programme has been traced back to the early 20th century from Hong Kong and has been rapidly and systematically developed over the last two decades in mainland China [30,31,32]. On the mainland, parenting, parental education, family education, and child-rearing programmes have been used interchangeably. Chinese scholars view family education from several perspectives. For example, family education has been colloquially referred to as parenting or parent education in the early stages of family life education and is an important part of family education [32,33,34]. Zhao [35,36] believes that parental education also exerts an influence on a child’s education. In this instance, family environment is the key to family education programmes. From the perspective of sociology, family education includes influences among family members and the social environment in which individuals are situated [34]. Although Chinese researchers have not reached a consensus on the definition of family education, they generally believe that family education is a complex and systematic process with the aim of not only increasing life skills, but also strengthening family members’ psychological and mental health, along with their well-being. This leads to an overall enhanced quality of life.

In China, historically, parents and family members have not received efficient evidence-based scientific support in child rearing [37]. Parents teach children by reinforcing the Confucian philosophy of the family, which was popular in ancient times. For example, “Zi bu jiao, fu zhi guo” exemplifies the attitudes of parents if their children make mistakes at home. This teaches that children’s mistakes are caused by a lack of parenting [34]. Another example is the Chinese parenting book called “Yan shi jia xun” (颜式家, AD 220-589), which is thought to be the first parenting book in history. It emphasises that the development of living skills and child rearing include love and strictness. Indulgence is not encouraged, and parents need to prioritise improving children’s morality via family education. Family education mainly focused on parenting in ancient times, i.e., parents taught children through modelling, with the purpose of fostering good characteristics, maintaining harmony, and maintaining the hierarchical structure of the family.

After the founding of the People’s Republic of China in 1949, family education was directed via government management, and related policies were proposed for the need to collectivise the nation [34]. Subsequently, women and children were kept in states of subordination. With the development of technology and the advancements to the economy, women’s voices have grown louder, and their family roles have shifted to become those of financially independent women; however, in some cultures, they are still expected to look after children and their elderly family members at home. According to this view, women have more responsibilities and family burdens in their nuclear families. To maintain harmony in the family, local communities and Women Federation offices have worked together to help mothers distribute information on parenting and strengthen family ties. This information is mainly based on Chinese cultural norms and family traditions, rather than the findings of scientific research [29,34]. Over the last two decades, service agencies and business sectors have tended to hold family education programmes and parent training by promoting research-led information. However, most of the research findings were applicable to Western contexts rather than a local Chinese context. As such, child-rearing information from Western research cannot be fully accepted with critical thoughts or without experiments applied to a local context.

To enhance Chinese parents’ knowledge and scientific skills in child rearing, the National People’s Congress voted the Family Education Promotion Law into effect at the end of 2021 [20]. The law advocates that social workers, regional family committees, educators, and researchers should work in an interdisciplinary team to provide scientific guidance and support to parents and families, for example, via family education workshops, parent training, and care for neglected children. All activities and programmes should improve children’s physical and mental development in order to enhance their education equity and quality [20]. This is extremely important in rural and disadvantaged areas, such as minority autonomous and mountainous regions.

The majority of the Inner Mongolia Autonomous Region is of Han nationality, with a sizeable Mongol minority of close to 5,000,000, which is the largest Mongolian population in the world (larger than that of Mongolia) [38]. Compared with other minority regions, Inner Mongolia has rich natural resources, such as non-ferrous-metallic mineral resources, coal mines, and sources of cashmere. All of this industry helps people in Inner Mongolia become the most economically developed provinces in China, with an annual GDP per capita of close to USD 13,000, often ranked as fifth in the nation [38]. Although the GDP of Inner Mongolia is ranked first in China, the quality of education has been far behind that of the Han ethnic majority.

Based on the enhancement of the Family Education Promotion Law, this explorative study focuses on developing a thorough understanding of parents’ knowledge, needs, and preferences for child-rearing family programmes in two large cities in the Inner Mongolia Autonomous Region (Ordos and Baotou). To achieve this, we attempted to answer the following two questions:

1. What knowledge of parenting and resources can be gained from family programmes in the Chinese Inner Mongolia Autonomous Region?
2. What are parents’ needs and preferences for family programmes in the Chinese Inner Mongolia Autonomous Region?

## 2. Instrument

This study used semi-structured interviews with open-ended questions, both face-to-face and via telephone. According to [39], though there are various methods that a researcher can use, however, “if the researcher’s goal is to understand the meaning people involved in education make of their experience, then interviewing provides a necessary… avenue of inquiry” (p. 10). In this study, interviewing was an appropriate method to use for investigating parents’ perceptions on their needs and preferences of family programmes in Inner Mongolia. In this way, it is better to improve parents’ knowledge and skills in caring children. Additionally, it could be helpful for educators to design family programmes.

## 3. Data Collection and Analysis

After obtaining ethics approval from the first author’s university, we sent the re-search information package to the education bureaus in two large cities in Inner Mongolia Autonomous Region to seek their support in recruitment. The research flyer, consent forms, and all research packages were individually sent to preschool managers in these two regions via registered information. Only participants who signed the consent forms were contacted by the research assistant to schedule their interview time (questions are available in the Appendix A protocols). All interviews were undertaken online due to pandemic travel restrictions. Semi-structured interviews were conducted and recorded by the principal researcher and research assistant. Each interview lasted approximately 40–45 min. The principal researcher then used the constructivist grounded theory approach [40] and NVivo software version 12 to transcribe and analyse the recordings.

We believe that constructivist grounded theory was appropriately utilised in the qualitative stage. First, it consists of “systematic inductive guidelines for collecting and analysing (sic) data to build middle-range theoretical frameworks that explain the collected data” [40]. Second, the researcher gathers and analyses the data, which are systematically “grounded” [41]. Third, the theory is generated and emerges from the raw data, which avoids the researcher’s presumptions and perceptions. The theory was generated in the process of comparatively analysing patterns, themes, and categories from participants’ responses within this body of research [42]. The researcher used a three-step coding approach within the constructivist grounded theory framework. Firstly, the researcher carefully read each line of the interview transcripts to identify the facts, comparing and then regrouping the lower categories with the higher sub-themes. Finally, after these processes, themes emerged from the subthemes [43].

### Participants

This study involved 24 participants from two large regions from Inner Mongolia. All participants had been living in Inner Mongolia for over 20 years, and their children were born in minority regions. All participants identified as Mongol nationals. Thirteen mothers and nine fathers from different families participated in the semi-structured interviews. For the individual families, participants were required to ensure that at least one child enrolled in preschool during the project period. Participants’ background information is presented in Table 1.

## 4. Results

The results and findings reported in this study are textual data retrieved from semi-structured interviews (See Table 2). The results can be classified into the following three key themes:

- The child’s safety and nutrition;
- The child’s physical and mental development skills;
- The child’s soft skills.

### 4.1. Theme 1: The Child’s Safety and Nutrition

Through this theme, the participants had a strong need and preference for knowledge and skills to improve child safety and nutrition. In addition, parents expressed that they made significant efforts to learn scientific ways of protecting their children, seeking a secure and enjoyable home environment, and improving children’s necessary nutrition for their mental and physical development. All parents believed that they received information from various resources, but they were not provided with enough support to identify practical guidance and scientific methods in real life. Discussions focus on how to provide a secure home and early years’ learning environment for children.

#### 4.1.1. Child’s Safety in Home and EC Environment

The participants in this study reported that they wanted to obtain information and improve their understanding of identifying a safe home and EC environment. Most mothers worried about their children’s safety if they lived in an unsafe environment in their early years. For example, a mother raised the following concerns:

“We live very close to a substation. We are not sure if the residential location would be appropriate for young children under six years [of age]. Some EC teachers told us that it should be fine for children to live near the highway, but they [were] not sure if the substation would be appropriate for children. You know, we cannot relocate in a short period, as the apartment is close to my husband’s workplace. Personally, I would like to learn more about the scientific information in the field.”

(Participant 12)

Aside from the choices of residential locations, another young mother (Participant 13) expressed her eagerness to know whether digital technologies, magnetic toys, and other electronic appliances would be harmful to children in the home. In particular, she was extremely worried about her children playing with magnetic toys for extended periods of time. However, she also related that she knew that children could not avoid being engaged in digital technologies in their daily lives but was unaware of how to choose a suitable product for her child. In this regard, she felt very unsatisfied that she was not given guidelines from family education programmes on how to choose appropriate digital technology for her children in a home environment.

All participants from Inner Mongolia emphasised that they had not been provided with sufficient parenting support to tailor their needs. “Our geographical location is unique, but there are [no] geologists to be invited into the family programme to sup-port us to identify which residential areas would be more appropriate for young children to live”. EC educators, teachers, and community workers cannot use their knowledge to guide parents. In terms of nutrition, both mothers and fathers have a strong interest in obtaining more scientific resources from interdisciplinary professionals and scholars in a local context. They are not satisfied with only being provided with a list of vitamins or paperwork from health professionals in EC centres. They doubt the validity of the data provided by non-scientific resources. A mother of two children felt extremely unsatisfied that she was often contacted by health professionals in EC teachers to ask her to buy health products and supplements for her children, as she did not know if her children were actually malnourished.

#### 4.1.2. Children’s Physical and Mental Development Skills

The successful development of children’s physical and mental development skills can be aided by a range necessary information regarding their different developmental stages. Parents generally show interest in obtaining information and scientific knowledge to support their children and enhance their language, literacy, and numeracy skills throughout their early years. These skills have been emphasised by participants as they are essential for children in future academic studies. Meanwhile, parents with male children showed a great interest in learning skills to improve their physical development, particularly those related to child-oriented physical activities, bio indexes, and their health conditions. Similar to the needs and preferences of children’s safety and nutrition, parents were not satisfied with the information provided by the EC teachers and educators. Parents stated that they had joined regular parent meetings and were told that they should pay attention to their children’s physical and mental development. However, there are no experts in family education programmes that can tailor advice to their needs. When asked to share their preferences in more detail, the fathers of the two children made the following comments:

“I am very appreciative [of the fact] that I was invited to participate in the inter-views [for] this project. You know, I am very busy in my daily job, and most of the time, my wife looked after my children. When I finished my job and returned home, they slept and might not have seen me the following morning. In this case, what I could communicate with them was from the notebook provided by the EC teachers. It is really difficult for me to be asked to attend all kinds of parenting programmes because EC teachers told me that my younger daughter should receive an intervention. I asked the EC teachers and educators why [she] needed to see the specialist, and was only told that teachers found that she was too shy to play with others. Without any other sup-port, I felt very disappointed with these parenting programmes.”

(Participant 24)

In addition to academic skills, parents are also interested in obtaining information on how to balance their children’s academic studies and social lives. Most parents prefer to be provided more information and skills in terms of reducing children’s stress and increasing their well-being via parenting programmes.

#### 4.1.3. Children’s Soft Skills

Compared with children’s academic skills, a small number of participants showed interest in enhancing their soft skills, including self-control, self-confidence, and peer relationships. Although the number of responses was small, the participants mentioned that a child’s soft skills cannot be measured in examinations, but they are helpful for children’s future success and life-long learning. For example, a mother shared with us her needs for the family education programme: “My son does not have good self-control as he was criticised by teachers [for] delaying handing in his homework. I need to learn skills to improve his self-control and self-discipline”. However, many parents commented that there was no information provided to strengthen their children’s soft skills. Parents were concerned about other aspects of children’s soft skills in terms of their peer relationships and child–parent relationships. These are extremely concerning for parents with two children. One father commented that he had little knowledge of how to develop peer relationships between the two children. He further explained:

“My parents and parents-in-law are [the] main carers [of] the two children. As the little one is a boy, my daughter always feels that grandparents love the little brother much more than her. When the two kids grabbed the same toys, the… grandparents always asked my daughter to give [them to] her little brother. Even when my daughter asked for support, my wife could not do anything. We believe that larger brothers and sisters should look after smaller siblings. This is our tradition.”

(Participant 17)

## 5. Discussion and Limitations

This paper highlights the vital role of family education programmes that should be tailored to provide efficient support for children’s development at all stages, including children’s security and nutrition, mental and physical development, and soft skills. The importance of knowing parents’ needs and preferences must be recognised before designing parent education family programmes. Parents have a good understanding of basic child-rearing skills, such as obtaining information from EC teachers, educators, and health professionals through parent meetings. In particular, if mothers are the main caregivers at home, they are more concerned about their child’s security and nutrition. The result is similar to that of Safadi et al. [22], who found that Jordanian mothers’ knowledge only focuses on children’s security and safety, lacking resources to support the development of children’s cognitive and interactive skills. Safadi et al. [22] emphasised that vulnerable mothers with lower levels of education paid more attention to children’s security. However, in this study, we did not intend to use a causal relationship to seek relationships between mothers’ professional and educational pedigrees and their skills in providing security for their children. We found that female participants of the same identity were the main caregivers looking after the children in the study.

Due to the unique location and rich natural resources in the two large cities in the Inner Mongolian Autonomous Region, participants were eager to obtain more knowledge and specific information that could be tailored to their family needs. This is reflected in the urgent need for scientific research to add reliable knowledge to the field of health and nutrition since many parents do not believe in the resources obtained by EC teachers and other business sectors. The participants perceived a negative experience as lacking scientific information about their preferences in parenting programmes and children’s daily lives. In addition, unique geographic factors can influence parents’ desires to know more about their children’s residential environment. The results echo the basis of the Family Education Promotion Law: interdisciplinary professionals and researchers need to work collaboratively to provide scientific guidance and support parents and children, via family education workshops, parent training, and community programmes [37]. Additionally, Yang et al. [34] found that the geographical locations of families are essential for parents to acquire scientific information, and consequently, their children might not develop as fast as their counterparts. Interdisciplinary research among various professionals and academics has also been encouraged via the ecological model [11]. Bronfenbrenner [11] explained the importance of how social environment and human interaction impact family and children. This may be extremely important for children with special educational needs. Howie [44], for example, discussed the application of Bronfenbrenner’s ecological theory in teaching young children, its utility in promoting inclusive educational environments through promoting school–family partnerships, and attending to the shared and individual needs of students from all ‘ecological niches’. In addition, in their life trajectories, children need various kinds of support, not only from parents but also from teachers, health professionals, educators, and specialists. In the human interaction process, parents could have a stronger partnership with these professionals and improve their understanding of their knowledge. Consequently, they might be more confident and positive about child rearing.

Although China’s family planning policies have ended, most parents still only have one child. While these parents are at work, grandparents take on a lot of responsibilities in looking after the children. Consequently, the traditional perspectives of parenting might go against modern perspectives. We found that parents had a mixed perspective of parenting in a traditional Chinese context. Some parents would like to use more evidence-based scientific methods in child rearing, which have not been addressed in previous research. Compared with the child-rearing methods of the last century, these parents have changed their attitudes towards traditional child-rearing methods employed by elderly members of the family. In addition, parenting is no longer driven by collectivism in the nation [29], and most women are financially independent. From this perspective, most parents have paid more attention to child rearing that is influenced by data from scientific research, rather than the daily experiences of older generations. However, in minority regions, many women are still housewives, and their main responsibilities are looking after their children and parents at home. These families rely heavily on external support as a major source of parenting knowledge and skills. In these families, senior parental authority plays a dominant role in the aims of parenting programmes.

In a similar vein, some participants were keen to improve their knowledge and skills by supporting their children’s academic skills rather than other soft skills. Even though they understand that children’s well-being is far more important than the scores in examinations, they are afraid that their children will not catch up with others of a similar school age. This and other needs are unique, as they are closely related to Chinese Confucianism, as discussed by scholars [29,34]. Lu [45] argued that children would be neither mentally nor physically healthy when studying for extended periods of time. This indicates that high-stake examinations push children to be a ‘learning machine’. However, some parents believe that children’s communication, self-control, and peer relationships are more important, and that these skills are important for parents to learn. The improvement in parents’ perceptions and preferences is closely related to economic and technological development in these two regions in the minority regions of Inner Mongolia. Yang [34] showed that Chinese family education programmes cannot be rapidly developed without financial support from local governments and NGOs. In this regard, parental needs and preferences in minority regions are not dynamic.

Although this study yielded interesting data, we cannot deny that it has three main limitations. Firstly, this study only used semi-structured interviews rather than a mixed research method, which would have identified participants’ perceptions related to their work and life experience in Inner Mongolia. In this case, it might be difficult to perform a triangulation of our findings. Second, this study is exploratory research that shows a case in one of the Chinese minority regions, and thus the sample size is small. However, there are five large minority autonomous regions where people might have different needs and preferences in family education programmes due to different cultures, religions, and languages. These potential cultural and historical factors should be considered in future research. Third, this study focuses on parents’ preferences and needs. In the near future, children and their family members should also be considered in regional studies. Their unique family background would form an interesting basis for field studies.

## 6. Conclusions

This study explored Chinese parents’ knowledge, preferences, and needs regarding the potential implementation of interdisciplinary child-rearing programmes in the Inner Mongolia region of China. The results showed that most participants held a positive attitude towards obtaining knowledge and skills in using evidence-based information to assist children’s mental and physical development. Interesting to know that parents often have mixed opinions on acquiring knowledge and skills to enhance children’s academic and soft skills in the context of traditional Chinese cultural norms. This might be due to Chinese traditional hierarchal family structures. Generally, younger parents held a positive attitude towards using a scientific method to develop their children’s mental and physical health.

We believe that Chinese family education programmes merit ongoing research. Future research could address the limitations of this study and undertake further investigations in the following ways.

(1) An interdisciplinary team should be built to design family education programmes based on Bronfenbrenner’s model [11]. Relevant workshops and training sessions could be conducted to improve the awareness of EC educators and community workers of the need to build positive partnerships with families and communities and to enhance their ability to do so. Such a study could identify whether the strategies proposed in the related research could support frontline educators and benefit parents. Such research would also support the development of more appropriate parenting programmes.
(2) Conduct a study involving variables related to family backgrounds, such as family ties, history, and life experience, to explore whether family heritage could affect parenting knowledge, needs, and preferences. Such a study could employ a longitudinal design based on the above suggestions, and thus determine how different parents perceive and behave in family education programmes. Such a study would provide information to authorities regarding how to tailor advice and support to families’ needs in different regions.

## 7. Implications

This study has important implications for parents, researchers, community workers, and EC educators. Stakeholders need to consider promoting family education pro-grammes through an enhanced understanding of both the external and internal factors associated with family needs and preferences. First, the findings show that parents in the minority regions of Inner Mongolia have basic knowledge of child rearing in children’s development. EC teachers and community workers might need to join regular PD programmes to improve their knowledge of children’s mental and physical development. This could be key for them to design and organise family education programmes. Second, frequent interactions are required between parents, researchers, and EC educators. Knowing parents’ needs and preferences is extremely important in being able to provide tailored and effective support for parents. Although teachers are extremely influential in young children’s lives, this does not suggest that educators should replace parents or that efforts to make changes at the macrosystem or exosystem level could be considered as being diminished. Rather, we urge the broader community to pay attention to the conditions experienced by, and qualities of, regional educators to promote the development of family education programmes and advocate for immediate social equity for children of venerable social statuses. Third, researchers should undertake more evidence-based scientific talks and parenting programmes to support parents who are keen to obtain first-hand data that support child rearing. Most importantly, the initiation of an interdisciplinary team needs to be considered at different levels of local strategic plans, such as local government, provincial, and national sectors, providing a holistic way to support Chinese families.

## Figures and Tables

**Table 1:** Participants’ demographic information.

Groups					
Mother	Age	Job Classification	Child(ren) Age	Family Income	Education Level
P1	30	Housewife	1.5 and 3 years old	CNY 50,000–100,000	BA
P2	32	Government officer	3 years old	CNY 50,000–100,000	MA
P3	27	Housewife	4 years old, 8 months	CNY 50,000–100,000	BA
P4	23	Self-employed	2 years old	CNY 50,000–100,000	Diploma
P5	29	B2B business	3 and 5 years old	CNY 100,000–200,000	BA
P6	30	Housewife	3.2 years old	CNY 50,000–100,000	BA
P7	48	Pre-school teacher	6 years old	CNY 50,000–100,000	Diploma
P8	43	Restaurant manager	5 years old	CNY 500,000–800,000	Diploma
P9	28	Self-employed	3.7 years old	CNY 50,000–100,000	BA
P10	29	Accountant	4 years old	CNY 50,000–100,000	BA
P11	32	School teacher	3.1 years old	CNY 50,000–100,000	BA
P12	34	Housewife	5.2 years old	CNY 50,000–100,000	BA
P13	29	Self-employed	6.3 years old	CNY 100,000–200,000	Certificate
Father					
P14	35	IT staff	1 and 3.4 years old	CNY 50,000–100,000	BA
P15	37	Civil officer	6 years old	CNY 50,000–100,000	BA
P16	29	Sales	3.2 years old	CNY 50,000–100,000	BA
P17	41	Self-employed	4 and 7 years old	CNY 100,000–200,000	MA
P18	27	Self-employed	4.1 years old	CNY 100,000–200,000	BA
P19	30	Driver	3.6 years old	CNY 100,000–200,000	certificate
P20	32	Engineer	1 and 4 years old	CNY 500,000–800,000	MA
P21	36	School teacher	4.5 years old	CNY 50,000–100,000	BA
P22	34	Businessman	2 years old, and 5 years old	CNY Over 1,000,000	BA
P23	29	Self-employed	4.3 years old	CNY 100,000–200,000	Diploma
P24	46	Family-owned business	3.3 years old and 10 years old	Over CNY 1,000,000	BA

**Table 2:** References obtained in the interviews.

Theses and Subthemes	References
Child’s safety and nutrition: Concerns on home environment; Concerns on preschool environment.	N = 68
	N = 43
	N = 25
Child’s mental and physical development: Academic issues; Behavioural issues; Confidence.	N = 57
	N = 33
	N = 17
	N = 7
Soft skills: Communication; Family relationships; Cultural values.	N = 44
	N = 26
	N = 12
	N = 6

## Data Availability

The data could be obtained by request from the authors. The data that support the findings of this study are available from the corresponding author, [JL], upon reasonable request.

## Appendix A

Semi-structured interview questions

1. How much do you know about your child or children in terms of mental and physical development? Where do you get this information?
2. How do you currently communicate and collaborate with EC teachers/families?
3. How important to you is the ability to obtain resources for your child or children’s development in the community/childhood centre?
4. What are your experiences of joining the parents’ family education programmes?
5. What skills and knowledge do you prefer to obtain as supportive information from family education programmes?
6. What do you need to know as a priority from family education programmes?
7. What are your views on future family education program design in the local context?
